# The duck plague virus UL7 protein mediates RIG-I degradation to block host antiviral responses and promote viral pathogenesis

**DOI:** 10.1016/j.psj.2025.105679

**Published:** 2025-08-12

**Authors:** Xing Lan, Yuan yuan Hao, Mingshu Wang, Linjiang Yang, Liping Wu, Anchun Cheng

**Affiliations:** a Institute of Veterinary Immunology and Green Drugs, Veterinary Department in College of Animal Science, State Key Laboratory of Green Pesticide, Guizhou University, Guiyang 550025, China; b Engineering Research Center of Southwest Animal Disease Prevention and Control Technology for ministry of Education of the People's Republic of China, International Joint Research Center for Animal Disease Prevention and Control of Sichuan Province, Key Laboratory of Animal Disease and Human Health of Sichuan Province, Research Center of Avian Disease and Institute of Veterinary Medicine and Immunology, College of Veterinary Medicine, Sichuan Agricultural University, Chengdu 611130, China

**Keywords:** Duck plague virus, UL7, Innate immunity, Viral life cycle, Pathogenicity

## Abstract

Duck plague (DP), which is caused by duck plague virus (DPV), is an acute, highly contagious disease with an extremely high mortality rate, and poses a serious threat to the waterfowl industry. DPV, which is an immunosuppressive virus, can significantly suppress host innate immune responses during the late stages of infection. However, the specific mechanisms by which the DPV UL7 protein functions in the viral replication cycle and immune evasion remain unclear. This study demonstrated that the DPV UL7 protein markedly inhibited IFN-β promoter activity, downregulated the transcription levels of IFN-β and downstream interferon-stimulated genes (ISGs), and suppressed the IFN-β promoter activity activated by effector molecules such as STING, cGAS, RIG-I, MDA5, MAVS, TBK1, and IRF7. Further research revealed that the UL7 protein directly interacted with RIG-I, inducing its degradation via the proteasome and lysosome pathways, thereby effectively inhibiting the host innate immune response. Although the UL7 protein was not involved in early infection processes such as viral adsorption, entry, or genome replication, it significantly promoted viral particle release and cell-to-cell spread, thereby increasing DPV proliferation *in vitro. In vivo* animal infection experiments further confirmed that compared with the parental virus DPV-CHv50 and the revertant virus DPV-UL7_null_ Rev, ducklings infected with the UL7 gene-deleted strain DPV-UL7_null_ presented significantly milder clinical symptoms, markedly reduced pathological damage in various organs, and a substantial decrease in mortality. These findings not only elucidate the molecular mechanism by which the UL7 protein mediates immune evasion by targeting RIG-I but also highlight its critical role in the pathogenicity of DPV.

## Introduction

Duck plague, which is commonly known as "big head disease" in China, is an acute, febrile and septicemic infectious disease caused by duck plague virus (DPV), and this disease affects ducks, geese and various Anseriformes species (Dhama et al., 2017). Mature DPV virions have a spherical morphology with a diameter ranging from 150–300 nm and are structurally composed of four concentric layers, which from the interior to the exterior, are the core, capsid, tegument and envelope (Spieker et al., 1996). The DPV genome is organized into four covalently linked regions, namely, the long unique region (UL), short unique region (US), internal inverted repeat (IRS) and terminal inverted repeat (TRS), which are arranged in the 5′-UL-IRS-US-TRS-3′ orientation (Chang et al., 2011). The characteristic clinical manifestations of DPV infection in affected ducks include ocular oedema, neck retraction and lacrimation. The pathological features of DPV infection include haemorrhagic lesions in the myocardium and liver, a grayish-yellow necrotic band at the oesophago-proventricular junction, haemorrhagic necrosis of the intestinal mucosa with button–like ulcers, lymphoid organ damage and degenerative changes in parenchymal organs (Aravind et al., 2015; Cai et al., 2023; El-Tholoth et al., 2019; Tang et al., 2024; Xuefeng et al., 2008). Although significant progress has been made in understanding the mechanisms underlying the pathogenesis of DPV infection, the regulatory networks and molecular mechanisms underlying host innate immune responses to viral infection remain poorly understood.

The innate immune system recognizes pathogen-associated molecular patterns (PAMPs) through pattern recognition receptors (PRRs) (Akira et al., 2006; Paludan & Bowie, 2013), and this recognition triggers type I interferon (IFN) antiviral responses that protect the host against pathogens (Kowalinski et al., 2011). The major classes of PRRs include Toll-like receptors (TLRs), NOD-like receptors (NLRs), retinoic acid-inducible gene-I-like receptors (RLRs), and DNA-sensing receptors (Hornung et al., 2006; Lee & Kim, 2007). RLRs, which are core members of the DExD/H-box RNA helicase family (Onoguchi et al., 2011), include three key components: retinoic acid-inducible gene-I (RIG-I), melanoma differentiation-associated gene 5 (MDA5), and laboratory of genetics and physiology gene 2 (LGP2) (Kowalinski et al., 2011). RIG-I can be activated by diverse RNA viruses, including both positive-strand (hepatitis C virus) and negative-strand RNA viruses (influenza virus, and Rift Valley fever virus) (Loo & Gale Jr., 2011). Notably, certain DNA viruses, such as HSV-1, may also activate RIG-I through indirect mechanisms (Chiang et al., 2018). Upon PAMP recognition, RLRs engage mitochondrial antiviral signalling proteins (MAVSs) through their caspase activation and recruitment domains (CARDs) (Belgnaoui et al., 2011). This interaction initiates a signalling cascade that activates TANK-binding kinase 1 (TBK1), which subsequently phosphorylates interferon regulatory factor 3 (IRF3) and IRF7. Then, the phosphorylated transcription factors dimerize and translocate to the nucleus to initiate type I IFN production. Secreted IFNs subsequently activate the JAK–STAT pathway to induce the expression of interferon-stimulated genes (ISGs). This leads to the production of effector proteins, such as myxovirus resistance protein (Mx), which has GTPase activity, and oligoadenylate synthetase-like (OASL) protein, ultimately establishing a broad-spectrum antiviral state (Gillich et al., 2023; Kato et al., 2006; Schoggins et al., 2011).

Significant progress has been made in functional research on the DPV genome, but the biological functions of the protein that is encoded by the *UL7* gene remain insufficiently elucidated. Studies on the functions of herpesvirus UL7 proteins have focused primarily on their interaction with UL51 proteins (Albecka et al., 2017; Fuchs et al., 2005; Pasdeloup et al., 2023; Schmitt & Keil, 1996), their involvement in the secondary envelopment of viral particles (Albecka et al., 2017; H. P. He et al., 2020), and their critical regulatory roles in viral replication (Fuchs et al., 2005; Shibazaki et al., 2020; Xu et al., 2016) and release (Fuchs et al., 2005; Schmitt & Keil, 1996). Although the DPV UL7 protein has been confirmed to modulate IFN-β promoter activity (Gao et al., 2022), the mechanisms underlying the role of the herpesvirus UL7 protein in immune evasion have not been reported. This study is the first to reveal that the DPV UL7 protein inhibits host innate immune responses by directly binding to RIG-I and mediating its degradation via the lysosomal and proteasomal pathways. Moreover, this study revealed that the *UL7* gene is nonessential for DPV replication, but the protein it encodes significantly increases the efficiency of viral proliferation *in vitro* by promoting viral particle release and cell-to-cell spread. Furthermore, the results of this study demonstrate that deletion of the *UL7* gene markedly reduces DPV pathogenicity in hosts. These groundbreaking findings not only expand the understanding of DPV UL7 protein functions but also provide new theoretical insights into the mechanisms underlying immune evasion by herpesviruses.

## Materials and methods

### Animals and ethics statement

The 9–11-day-old duck embryos used in this study were purchased from a farm in Ya'an city, Sichuan, China. One-day-old healthy ducklings were provided by the same farm and were negative for anti-DPV antibodies. The ducklings were housed in the animal experimental facilities of Sichuan Agricultural University in Ya'an, Sichuan Province, China. This study was approved by the Experimental Operation Guidelines and Animal Welfare Committee of Sichuan Agricultural University (approval licence no. XF20230122).

### Cells, viruses, reagents and antibodies

Duck embryonic fibroblasts (DEFs) were isolated from 9–11-day-old duck embryos and cultured in Eagle's minimum essential medium (MEM) (Gibco, Shanghai, China) supplemented with 10 % newborn bovine serum (Gibco, MD, USA) at 37°C in 5 % CO₂. The virulent Chinese strain of DPV (DPV-CHv) (GenBank accession number: JQ647509.1) and its 50th DEF-adapted passage (DPV-CHv50) were preserved and provided by the Institute of Veterinary Immunology, College of Veterinary Medicine, Sichuan Agricultural University. Poly(I:C) was purchased from InvivoGen (Cat: tlrl-pic), and the proteasome inhibitor MG132 and the autophagy lysosomal inhibitor chloroquine (CQ) were obtained from Selleck. Commercial antibodies, including rabbit anti-Flag, mouse anti-HA, and rabbit anti-beta (β)-actin antibodies, as well as goat anti-mouse and goat anti-rabbit secondary antibodies, were obtained from Proteintech (Wuhan, China). The rabbit anti-UL7 polyclonal primary antibody was provided by our laboratory, and Alexa Fluor-conjugated goat anti-rabbit IgG (Cat: A11008) was purchased from Thermo Fisher Scientific.

### Plasmids

The eukaryotic expression plasmids pCAGGS, UL7-HA, UL2-3HA, cGAS-Flag, STING-Flag, TBK1-Flag, IRF7-Flag, RIG-I-3Flag, MDA5-Flag, MAVS-Flag, IFN-β-Luc, and pRL-TK were preserved and provided by the Institute of Veterinary Immunology, College of Veterinary Medicine, Sichuan Agricultural University. The primers used in this study are listed in Table 1.

**Table 1:** Sequences of all primers and probes used in this study.

Primer name	Primer sequences	Gene
ΔUL7-Kan-F	GGTTTACAGGCGCCTCAGTCATGAGTCTGAGGGTAAATAGTGGGGATAACAGGGTAATCGATTT	∆UL7Kan-targeting fragment
ΔUL7-Kan-R	GAATAAATTGTATTTTTTATTTGTTTCATAAACAACTAAGCTATTTACCCTCAGACTCATGACTGAGGCGCCTGTAAACCGCCAGTGTTACAACCAAT	
UL7 Rev F	GGTACCAGGCATCCCTACGCGGTTTACAGGCGCCTCAGTCATGAGTCTGAGGGTAAATAGTAATAACCTACAACAGTTAT	UL7 targeting fragment
UL7 Rev R	TTTATTTGTTTCATAAACAACTAAGTTAGTATGTATAATAAAGCT	
∆UL7-Kan Rev F	CTTAGTTGTTTATGAAACAAATAAAAAATACAATTTATTCATTTGTGTCTTAGGGATAACAGGGTAATCGAT	Kan fragment containing left and right homologous arms of UL7
∆UL7-Kan Rev R	TCACACTAATATTTGAAATAAAACGTTTGAAGACACAAATGAATAAATTGTATTTTTTATTTGTTTCATAAACAACTAAGTGTTACAACCAATTAACC	
∆UL7 F	ATGGATATGAACCAGAGC	Identification of the UL7 targeting fragment
∆UL7 R	GTTCCATATTATCGCACG	
∆Kan R	CCTGAGCGAGACGAAATACG	
cGAS F	CTACTACGAGCGCGTCAAGA	cGAS
cGAS R	CTGAATCCTCGCGATAGGCA	
TBKI F	TTAGAGGAGCCATCCAACGC	TBKI
TBKI R	AGTTCTCTCGCAGCACCAAA	
IRF7 F	AACGCCAGGAAGGATGTCAC	IRF7
IRF7 R	CGCAGCGAAAGTTGGTCTTC	
INF-β F	TCTACAGAGCCTTGCCTGCAT	IFN-β
INF-β R	TGTCGGTGTCCAAAAGGATGT	
RIG-I F	TATGACCCTCCCAAGCCAGA	RIG-I
RIG-I R	GCAATGAGCAGCCTGTTGTC	
MAVS F	AGCCCAGAAATGAACCCCAG	MAVS
MAVS R	TCGAACTGCTGCTGGATGAG	
OASL F	TCTTCCTCAGCTGCTTCTCC	OASL
OASL R	ACTTCGATGGACTCGCTGTT	
MX F	TGCTGTCCTTCATGACTTCG	Mx
MX R	GCTTTGCTGAGCCGATTAAC	
IL-6 F	TTCGACGAGGAGAAATGCTT	IL-6
IL-6 R	CCTTATCGTCGTTGCCAGAT	
18S rRNA F	GTACAGTGAAACTGCGAATGG	18S rRNA
18S rRNA R	CGTCGGCATGTATTAGCTCTA	

### Dual-luciferase reporter assay

The luciferase reporter plasmid IFN-β-Luc, the internal control plasmid pRL-TK, and specific expression plasmids were cotransfected into DEFs. After 36 h of transfection, the cells were collected and lysed with lysis buffer to prepare samples. To analyse changes in IFN-β promoter activity, the activities of firefly luciferase and Renilla luciferase were measured according to the instructions of the TransDetect® Double-Luciferase Reporter Assay Kit (TransGen Biotech).

### RNA extraction and RT‒qPCR

Total RNA was extracted from treated samples with RNAiso Plus Reagent (TaKaRa) and reverse transcribed into cDNA with the PrimeScript™ RT reagent kit (Takara, Japan). The mRNA expression levels of various cytokines in cells were quantified with a SYBR® Premix Ex Taq™ II (Tli RNaseH Plus) kit (Takara), and 18S rRNA served as the endogenous reference gene. Relative gene expression was calculated by the 2^−ΔΔCt^ method. The sequences of the primers used for real-time quantitative PCR are listed in Table 1.

### *Western blotting analysis*

The cells were washed with PBS and lysed with RIPA buffer supplemented with protease inhibitors on ice for 30 min, after which the samples were centrifuged at 4°C for 15 min to collect the supernatants. Protein samples were denatured by boiling in 5× SDS loading buffer, separated by SDS‒PAGE, and transferred to PVDF membranes (preactivated with methanol for 30 s) at 220 mA in an ice bath. After being blocked with 5 % skim milk for 2 h, the membranes were incubated with primary antibodies overnight at 4°C (or for 2 h at room temperature), washed with TBST, and then incubated with HRP-conjugated secondary antibodies (1:3000) for 1 h at room temperature. Protein signals were visualized with an enhanced chemiluminescence (ECL) detection kit (Takara, Japan).

### Coimmunoprecipitation (Co-IP)

After 36 h of transfection, the cells were collected and washed with ice-cold PBS, followed by lysis with IP lysis buffer on ice for 30 min. The lysates were centrifuged at 4°C for 15 min, and 50 μL of the supernatants was used for protein expression analysis. The remaining lysates were incubated with a specific antibody at 4°C overnight. Subsequently, BeyoMag™ Protein A+G magnetic beads (Beyotime, Shanghai, China) were added and incubated at room temperature for 1 h. Finally, the bead complexes were washed with PBS, resuspended in 5× loading buffer, boiled, and subjected to Western blotting analysis.

### *Inhibition of protein degradation pathways*

The UL7-HA and RIG-I-3Flag plasmids were cotransfected into DEFs, and the following negative controls were established: cotransfection of pCAGGS and RIG-I-3Flag, cotransfection of DMSO and RIG-I-3Flag, and nontransfection of UL7-HA. After 24 h, the cells were treated with 20 µM/mL MG-132 or 100 mM/mL CQ together with 100 µg/mL cycloheximide (CHX) for 8 h. Cell samples were then collected and subjected to Western blot analysis.

### Indirect immune fluorescence assay (IFA)

After being washed with PBS, the cells were fixed with 4 % paraformaldehyde at 4°C for 12‒16 h and then permeabilized with 0.25 % Triton X-100 at 4°C for 30 min. To minimize nonspecific binding, the cells were blocked with 5 % bovine serum albumin (BSA) at 37°C for 2 h. The samples were then incubated overnight at 4°C with a rabbit anti-UL7 polyclonal primary antibody (1:100), followed by incubation with an Alexa Fluor-conjugated goat anti-rabbit IgG secondary antibody (1:1000) at 37°C for 1 h in the dark. After the nuclei were counterstained with DAPI for 10 min, fluorescence images were captured with an inverted fluorescence microscope.

### *Establishment of DPV-UL7_null_ and DPV-UL7_null_ Rev strains*

In this study, we successfully constructed *UL7* gene deletion and revertant mutants with the bacterial artificial chromosome (BAC)-based DPV gene editing platform, following previously described methods (Wu et al., 2022). To establish the rescue recombinant virus, plasmids from *UL7* deletion and revertant infectious clones were extracted and transfected into DEFs. When fluorescence (EGFP) and cytopathic effects (CPEs) became visible in the transfected cells, the cell supernatant was collected and stored as viral stock. After purification and verification, the correctly identified *UL7* gene deletion and revertant strains were designated DPV-UL7_null_ and DPV-UL7_null_ Rev, respectively. Information about the primers used in this study is provided in Table 1.

### Restriction fragment length polymorphism (RFLP)

The corresponding infectious clone plasmid was extracted. The restriction digestion system (25 µL) consisted of 1 µg of plasmid, 1 µL of QuickCut™ EcoRI/KpnI, 2 µL of 10× QuickCut Buffer, and ddH_2_O to a total volume of 25 µL. Digestion was performed at 37°C for 2 h. Then, nucleic acid gel electrophoresis was performed as follows. A 1 % nucleic acid gel was electrophoresed at a constant voltage of 50 V for 4 h. After electrophoresis, the gel was visualized and images were captured with a gel imaging system.

### In vitro growth kinetics of DPV-UL7_null_

This study involved the use of two modes of infection to evaluate viral replication kinetics: a multistep growth curve (MOI of 0.01) and a one-step growth curve (MOI of 1). DEFs were infected with DPV-UL7_null_, DPV-UL7_null_ Rev, or DPV-CHv50. After 2 h of adsorption, the inoculum was replaced with maintenance MEM supplemented with 2 % newborn bovine serum (NBS). For multistep growth analysis, samples were collected at 24, 48, 72, and 96 h post infection (hpi). For one-step growth analysis, samples were collected at 6, 12, 24, 30, and 36 hpi. Viral titres were determined by a 50 % tissue culture infectious dose (TCID_50_) assay. Simultaneously, viral genome copy numbers were quantified by the TaqMan MGB probe-based qPCR method established by Guo (Weingarten, 1989), which involves the use of UL30-F/R primers and a DPV-specific probe.

### Virus adsorption, invasion, genome replication, and release assays

***Adsorption assay.*** DEFs were seeded in 12-well plates and prechilled at 4°C for 2 h. After being washed with ice-cold sterile PBS, the cells were infected with DPV-UL7_null_, DPV-UL7_null_ Rev, or DPV-CHv50 at an MOI of 0.01. After 2 h of incubation at 4°C, the supernatants were removed, the cells were washed five times with ice-cold sterile PBS and then collected, after which the viral genomes were quantified. ***Invasion assay.*** After virus adsorption (as described above), the cells were washed with ice-cold sterile PBS and maintained in 2 % NBS-supplemented medium. The cells were then incubated at 37°C for 1 h. After five washes with ice-cold sterile PBS, the cells were harvested, and viral copy numbers were determined. ***Genomic replication assay.*** DEFs were infected with the three viral strains at an MOI of 0.01. After 6 h, the medium was replaced with 2 % NBS maintenance medium. The cells were collected at 7, 8, 9, and 10 hpi, and the viral genomes were quantified. ***Release assay.*** DEFs were infected with three viral strains at an MOI of 1. After the cells were incubated for 18 h, the supernatants were discarded, the cells were washed with PBS, and the medium was replaced with 2 % NBS maintenance medium. The supernatants were collected at 1 h, 2 h, 3 h, and 4 h after the medium was changed, and the TCID_50_ was measured.

### *Viral plaque assay*

Preparation of DEFs in 6-well plates: DEFs were infected with DPV-UL7_null_, DPV-UL7_null_ Rev, or DPV-CHv50 at an MOI of 10⁻⁴. The cells were incubated in a cell culture incubator for 2 h, and then, the plates were gently shaken every 30 min. The supernatants were discarded, and the samples were washed with PBS. MEM supplemented with 1.5 % methylcellulose (Solarbio, Beijing, China) was added, and the plates were cultured in an incubator for approximately 5 days. After the medium was discarded, the cells were washed 3 times with PBS, 500 µL of precooled 4 % paraformaldehyde was added, and the mixture was incubated for 30 min at 4°C to fix the cells. The samples were washed 3 times with PBS, stained with 500 µL of 0.5 % crystal violet (Solarbio, Beijing, China) for 5 min, and washed under running water to remove the staining solution. Finally, the plaques were observed and counted.

### Viral infection experiment in ducklings

Seventy-six healthy 14-day-old ducklings were randomly divided into four groups (the DPV-UL7_null_ group, DPV-UL7_null_ Rev group, DPV-CHv50 group, and MEM control group), with 19 ducklings per group. Each group was intramuscularly injected into the leg with 1 mL of the corresponding viral suspension (10^6^ TCID_50_/mL) or MEM maintenance medium. During the experiment, the body temperature, weight changes, and survival rates of 10 ducklings per group were monitored and recorded daily. The remaining nine ducklings in each group were randomly selected and humanely euthanized at 3, 6, and 9 days postinfection (dpi). After dissection, gross lesions in the tissues and organs were observed and recorded. Organ samples were collected and subjected to histopathological sectioning and microscopic examination. Additionally, the viral load was determined by TaqMan probe-based quantitative real-time PCR (qPCR) to evaluate viral replication dynamics in the ducklings.

### Statistical analysis

All the statistical analyses were performed with GraphPad Prism 8.0 software. Intergroup comparisons were conducted by one-way analysis of variance (ANOVA), with statistical significance defined as follows: *P < 0.05, **P < 0.01, ***P < 0.001, and ****P < 0.0001; ns (not significant) indicates P > 0.05.

## Results

### The DPV UL7 protein suppresses poly(I:C)-and poly(dA:dT)-induced IFN-β production

**Fig. 1:**
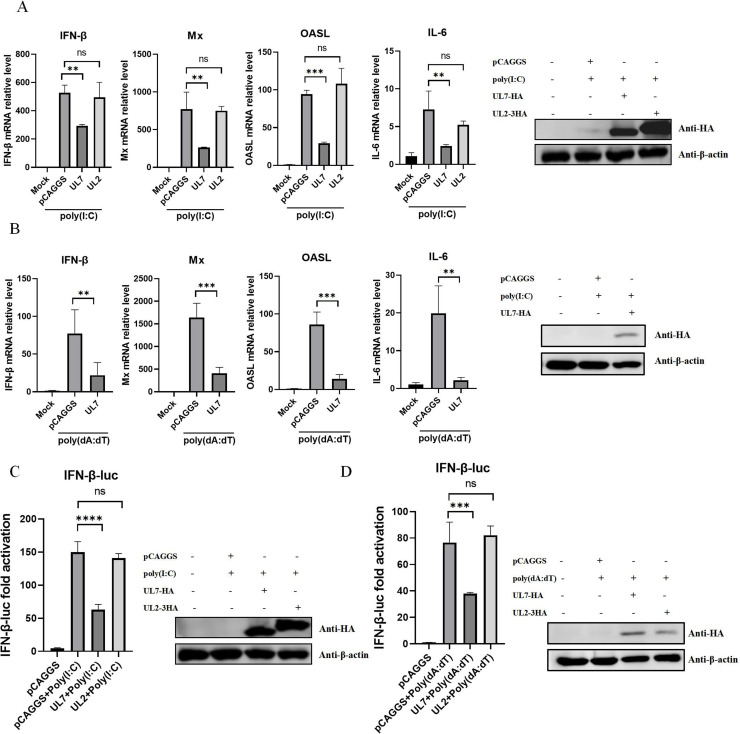
The DPV UL7 protein inhibits poly(I:C)-induced IFN-β production. (A) DEFs were transfected with UL7-HA, UL2-3HA, or empty vector, followed by poly(I:C) transfection 24 h later. The cells were harvested 12 h after poly(I:C) transfection. The mRNA expression levels of immune-related genes (IFN-β, Mx, OASL, and IL-6) were measured by RT‒qPCR, and the protein expression of UL7 and UL2 was measured by Western blotting. (B) DEFs were transfected with UL7-HA or empty vector, followed by poly(dA:dT) transfection 24 h later. The cells were harvested 12 h after poly(dA:dT) transfection. The mRNA expression levels of immune-related genes (IFN-β, Mx, OASL, and IL-6) were measured by RT‒qPCR, and the protein expression of UL7 was measured by Western blotting.(C) DEFs were cotransfected with UL7-HA, UL2-3HA, or an empty vector together with the reporter plasmid IFN-β-Luc and the internal control plasmid pRL-TK. After 12 h of transfection, the cells were stimulated with poly(I:C) for an additional 12 h before being harvested. IFN-β promoter activity was analysed using a dual-luciferase reporter assay system, and UL7/UL2 protein expression was examined by Western blotting.(D) DEFs were cotransfected with UL7-HA, UL2-3HA, or an empty vector together with the reporter plasmid IFN-β-Luc and the internal control plasmid pRL-TK. After 12 h of transfection, the cells were stimulated with poly(dA:dT) for an additional 12 h before being harvested. IFN-β promoter activity was analysed using a dual-luciferase reporter assay system, and UL7/UL2 protein expression was examined by Western blotting. The data were analysed by one-way ANOVA, **P < 0.01, ***P < 0.001; ns indicates no significant difference.

To elucidate the mechanism underlying the immunomodulatory effect of the UL7 protein, this study first investigated its regulatory effect on IFN-β production. DEFs were transfected with UL7-HA, UL2-3HA, or empty vector plasmids, followed by immune stimulation with the double-stranded DNA analo gue poly(dA:dT) or the double-stranded RNA analo gue poly(I:C). RT‒qPCR analysis revealed that the UL7 protein significantly suppressed the mRNA expression levels of IFN-β, OASL, Mx, and IL-6 induced by both poly(I:C) and poly(dA:dT), whereas the UL2 protein had no significant inhibitory effect under poly(I:C) stimulation (Figure 1A‒B). To further validate these findings, we cotransfected DEFs with the IFN-β-Luc reporter plasmid, the internal control plasmid pRL-TK, and either UL7-HA or UL2-3HA, followed by stimulation with poly(I:C) or poly(dA:dT) to measure luciferase activity. Consistent with the qPCR results, the IFN-β promoter activity in UL7-expressing cells was markedly lower than that in control cells, while UL2 expression had no significant effect (Figure 1C‒D). These data indicate that the UL7 protein specifically inhibits both the production of IFN-β and the expression of its downstream effector molecules in DEFs.

### The DPV UL7 protein inhibits IFN-β promoter activity via the cGAS-STING and RIG-I/MDA5 signalling pathways

**Fig. 2:**
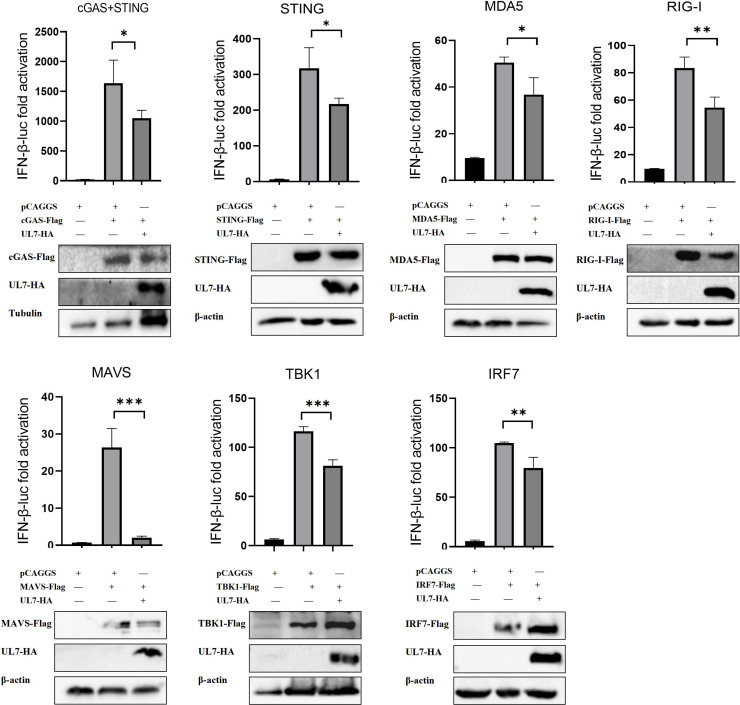
The DPV UL7 protein suppresses IFN-β promoter activity via the cGAS-STING and RIG-I/MDA5 signalling pathways. UL7-HA or empty vector was cotransfected with plasmids carrying cGAS, STING, MDA5, RIG-I, MAVS, TBK1, or IRF7 into DEFs. IFN-β promoter activity was measured using a dual-luciferase reporter assay, and protein expression levels were measured by Western blotting. The data were analysed by one-way ANOVA, *p < 0.05, **p < 0.01, and ***p < 0.001.

To elucidate the specific mechanism by which the UL7 protein suppresses IFN-β production, we further investigated its effect on IFN-β activation mediated by a DNA-sensing pathway (cGAS-STING) and RNA-sensing pathway (RIG-I/MDA5). In this experiment, DEFs were cotransfected with plasmids carrying key adaptor proteins (including cGAS, STING, TBK1, IRF7, RIG-I, MDA5, and MAVS), the IFN-β-Luc reporter plasmid, the pRL-TK internal control plasmid, and either the empty vector or the UL7-HA plasmid. IFN-β promoter activity was then measured using a dual-luciferase reporter assay system. The UL7 protein significantly suppressed the IFN-β promoter activity induced by all the tested adaptor molecules, and particularly pronounced inhibitory effects on RIG-I-, MAVS-, TBK1-, and IRF7-induced IFN-β promoter activation were observed (Fig. 2). These findings indicate that the DPV UL7 protein can simultaneously inhibit the IFN-β promoter activity induced by both the cGAS-STING and RIG-I/MDA5 signalling pathways, with more prominent inhibitory effects observed on the RIG-I-MAVS signalling pathway.

### The DPV UL7 protein inhibits RIG-I protein expression

**Fig. 3:**
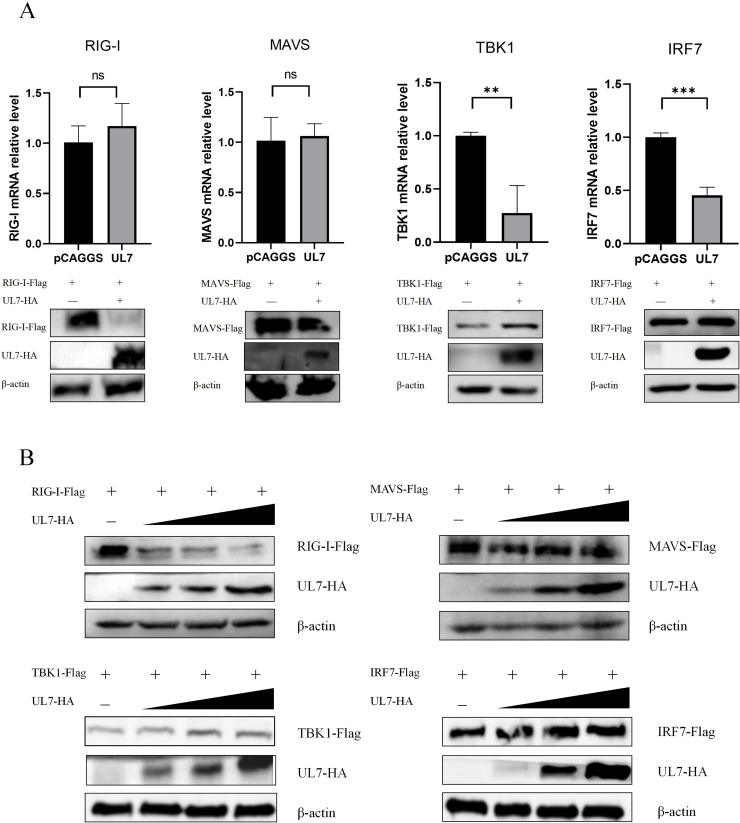
The DPV UL7 protein suppresses RIG-I expression. (A) The pCAGGS and UL7-HA plasmids were cotransfected with the TBK1-Flag, IRF7-Flag, RIG-I-3Flag, and MAVS-Flag plasmids into DEFs. After 36 h of transfection, the cells were collected, and total RNA was extracted. After reverse transcription to obtain cDNA, RT‒qPCR was performed to measure the expression levels of relevant genes. (B) A gradient dose design was used: pCAGGS (600, 400, 200, or 0 ng) and UL7-HA (0, 200, 400, or 600 ng) were cotransfected with a fixed dose (400 ng) of the TBK1-Flag, IRF7-Flag, RIG-I-3Flag, and MAVS-Flag plasmids into DEFs. After 36 h of transfection, the cells were lysed on ice in RIPA lysis buffer to obtain protein samples, after which target protein expression was measured by Western blotting. The data were analysed by one-way ANOVA, **P < 0.01, ***P < 0.001; ns indicates no significant difference.

On the basis of the experimental results described above, the current literature was considered; studies on the cGAS–STING signalling pathway are relatively extensive, whereas exploration of the RIG-I–MAVS signalling pathway remains limited. Therefore, we further investigated the mRNA and protein expression of key adapter proteins in the RIG-I-MAVS signalling pathway. DEFs were cotransfected with the empty vector or UL7-HA along with plasmids carrying eukaryotic RIG-I, MAVS, TBK1, and IRF7, and the transcription levels of target genes were measured by RT‒qPCR. The results revealed that UL7 protein expression significantly decreased the mRNA expression of TBK1 and IRF7 but had no obvious effect on the mRNA expression of RIG-I and MAVS (Fig. 3A). We further cotransfected DEFs with empty vector (600 ng, 400 ng, 200 ng, or 0 ng), UL7-HA (0 ng, 200 ng, 400 ng, or 600 ng), and plasmids carrying eukaryotic RIG-I, MAVS, TBK1, and IRF7 (400 ng), after which the resulting cell lysates were subjected to Western blotting analysis to measure target protein expression. The results demonstrated that as the protein expression level of UL7 increased, the protein expression of RIG-I gradually decreased, whereas the protein expression levels of MAVS, TBK1, and IRF7 did not significantly change (Fig. 3B). These findings suggest that the UL7 protein may specifically regulate RIG-I expression, thereby influencing the activity of the RIG-I-MAVS signalling pathway. This discovery provides new insights into the mechanistic role of the UL7 protein in innate immune regulation.

### The DPV UL7 protein interacts with RIG-I and reduces RIG-I protein levels via the proteasome and lysosome pathways

**Fig. 4:**
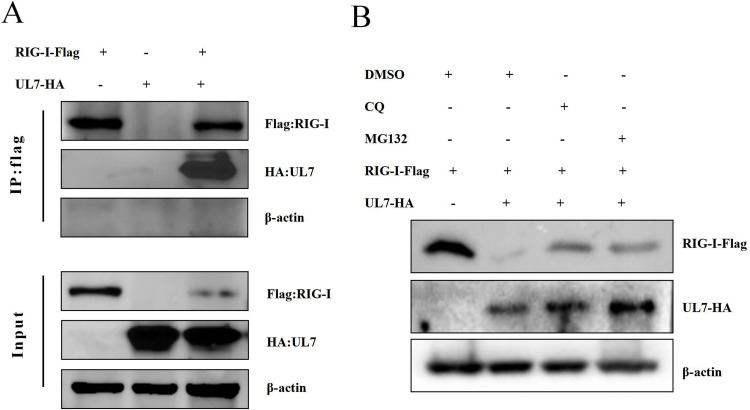
The DPV UL7 protein interacts with RIG-I and promotes its degradation through proteasomal and lysosomal pathways. (A) DEFs were transfected with UL7-HA and RIG-I-3Flag plasmids either individually or in combination. At 36 h posttransfection, the cells were lysed with IP lysis buffer on ice, and the lysates were subjected to Western blot analysis. (B) DEFs were cotransfected with UL7-HA and RIG-I-3Flag plasmids, and a negative control group (without UL7-HA transfection) was included. After 24 h of transfection, the cells were treated with 20 µM MG-132 or 100 mM CQ in combination with CHX for 8 h, followed by Western blotting analysis.

The UL7 protein significantly inhibited RIG-I protein expression without affecting RIG-I mRNA levels. To elucidate the mechanism underlying this regulatory effect, we first investigated the interaction between UL7 and RIG-I. Coimmunoprecipitation (co-IP) assays confirmed that UL7-HA directly interacted with RIG-I in DEFs cotransfected with the respective plasmids (Fig. 4A). Because UL7 did not alter RIG-I mRNA levels, we hypothesized that it might regulate RIG-I protein stability through degradation pathways. In eukaryotic cells, protein degradation occurs via the ubiquitin‒proteasome and autophagy‒lysosome pathways. To test this hypothesis, DEFs were treated with the proteasome inhibitor MG132 or the lysosome inhibitor CQ, and protein synthesis was blocked with CHX. Both inhibitors significantly alleviated UL7-mediated RIG-I protein degradation, whereas no notable changes were observed in the control group (Fig. 4B). These findings indicate that UL7 promotes RIG-I degradation through both the proteasome and lysosome pathways.

### UL7 protein deficiency inhibits DPV proliferation

**Fig. 5:**
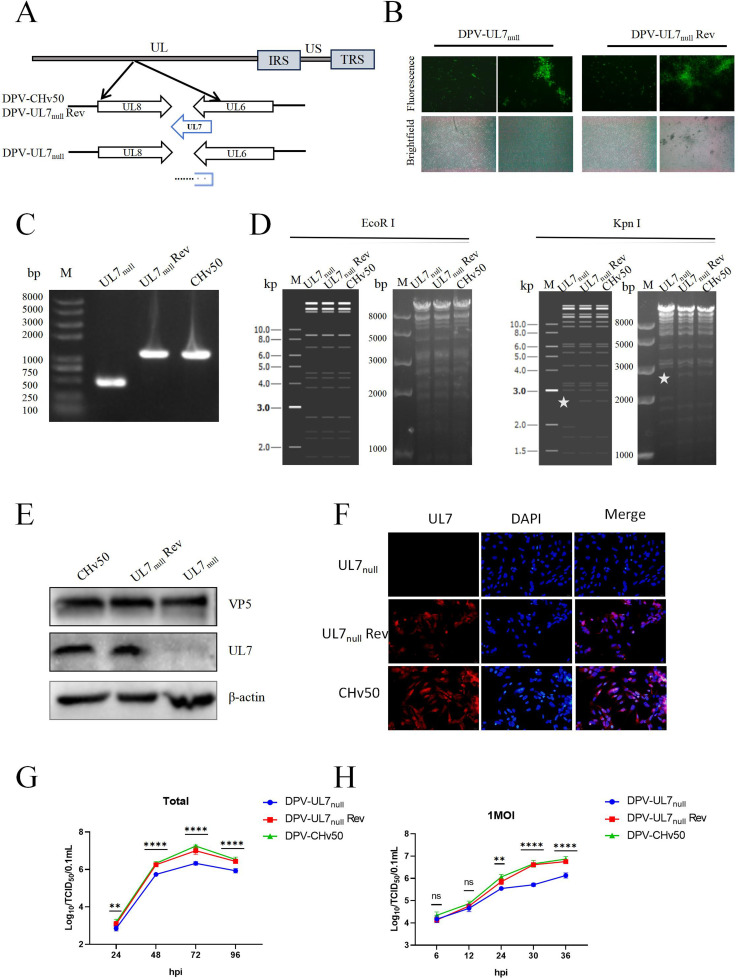
Generation and characterization of DPV-UL7_null_ virus revealing the role of UL7 in viral replication. A specific deletion of amino acid residues 90–321 in the UL7 protein (a nonoverlapping region between UL7 and UL6) was introduced into the DPV genome, and a corresponding revertant mutant was constructed. (A) The constructed UL7-deficient and revertant viral infectious clones were transfected into DEFs to prepare the viruses. The successfully prepared deletion and revertant viruses were designated DPV-UL7_null_ and DPV-UL7_null_ Rev, respectively (B). The recombinant viruses were systematically verified by PCR (C), RFLP (D), WB (E), and IFA (F). DEFs were infected with DPV-UL7_null_, DPV-UL7_null_ Rev, or DPV-CHv50 at multiplicities of infection (MOIs) of 0.01 and 1. Viral samples were collected at the indicated time points, and viral titres were determined to plot multistep (G) and one-step (H) growth curves. The data were analysed by one-way ANOVA, **P < 0.01, ****P < 0.0001, ns indicates no significant difference.

The experiments described above confirmed that the DPV UL7 protein facilitated the degradation of the RIG-I protein through both the proteasomal and lysosomal pathways, thereby effectively suppressing IFN-β signalling pathway activation. Because the type I interferon response is a key host defence mechanism that restricts viral replication, we hypothesized that UL7-mediated immune evasion may directly promote viral proliferation. To test this hypothesis, a UL7-deficient virus (DPV-UL7_null_), which lacks amino acids 90–321 (the region of the *UL7* gene that does not overlap with the UL6 gene) (Fig. 5A) and a revertant virus (DPV-UL7_null_ Rev) were constructed and purified to yield fluorescence-free viruses (Fig. 5B). Multiple validation methods were used: the PCR products of DPV-UL7_null_ had the expected size of 462 bp, whereas DPV-UL7_null_ Rev and the parental virus DPV-CHv50 yielded PCR products of 1156 bp; these results were consistent with the theoretical predictions (Fig. 5C). RFLP analysis revealed that EcoR I-digested pDPV-UL7_null_ produced bands that matched expectations, and KpnI digestion resulted in pDPV-UL7_null_ lacking a 3-kb band compared with pDPV-UL7_null_ Rev and pDPV-CHv50; these results were consistent with the simulated outcomes (Fig. 5D). WB and IFA results confirmed that the UL7 protein was absent only in DPV-UL7_null_ cells (Fig. 5, Fig. 5). These results verified the successful construction of the DPV-UL7_null_ and DPV-UL7_null_ Rev strains, enabling subsequent functional studies.

Next, the impact of the UL7 protein on viral proliferation was evaluated through multistep and one-step growth kinetic experiments. The results (Fig. 5, Fig. 5) demonstrated that compared with DPV-UL7_null_ Rev and DPV-CHv50, DPV-UL7_null_ exhibited significant proliferation defects in terms of both growth kinetics, with markedly reduced viral titres and genome copy numbers. The proliferation kinetics of DPV-UL7_null_ Rev were nearly identical to those of DPV-CHv50, indicating that *UL7* gene restoration effectively restored the viral proliferative capacity. These data conclusively demonstrate that the UL7 protein plays an indispensable role in promoting the proliferation of DPV *in vitro*.

### UL7 protein deficiency inhibits DPV release and cell-to-cell spread

**Fig. 6:**
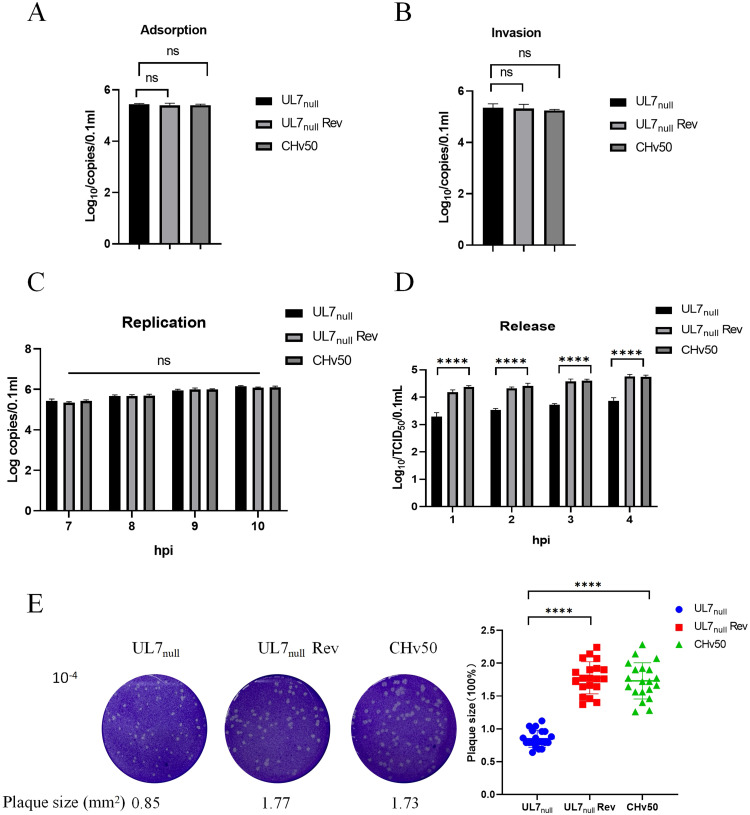
UL7 protein deficiency inhibits DPV release and cell-to-cell spread. DEFs were infected with DPV-UL7_null_, DPV-UL7_null_ Rev, or DPV-CHv50 at an MOI of 0.01 at 4°C. After 2 h of viral adsorption, the cells were collected, and viral copy numbers were determined by qPCR to analyse viral adsorption efficiency. (A) After adsorption, the cells were incubated at 37°C for 1 h. The cells were then lysed, and the number of intracellular viral genome copies was measured to assess viral entry efficiency. (B) DEFs were infected with the three virus strains at an MOI of 0.01. At 6 h postinfection (hpi), the medium was replaced, the cells were collected at 7, 8, 9, and 10 hpi, and the number of viral genome copies was determined to assess replication efficiency. (C) DEFs were infected with the three virus strains at an MOI of 1. At 18 hpi, fresh medium was added. The supernatants were collected at 1, 2, 3, and 4 h after the medium was changed, and the viral titres were determined by plaque assays to evaluate viral release efficiency. (D) DEFs were infected with the three virus strains at MOIs of 10^−4^. Plaque formation was visualized by crystal violet staining, and 20 randomly selected plaques were statistically analysed. (E) The data were analysed by one-way ANOVA, ****P < 0.0001; ns indicates no significant difference.

To further investigate the role of the UL7 protein in the DPV lifecycle, we analysed its functions at key stages of the viral lifecycle, including adsorption, invasion, genome replication, release, and cell-to-cell spread. The experimental results revealed no significant differences among the three viral strains in terms of adsorption (Fig. 6A), invasion (Fig. 6B) or genome replication (Fig. 6C). However, the absence of *UL7* significantly reduced viral release efficiency (Fig. 6D) and cell-to-cell spread (Fig. 6E), whereas the revertant virus restored both viral release efficiency and cell-to-cell spread to levels that were comparable to those of the parental virus. In conclusion, these findings demonstrate that the UL7 protein primarily increases DPV proliferation by promoting viral release and cell-to-cell spread rather than affecting early infection processes or genome replication.

### DPV UL7 protein deficiency increases immune responses in DEFs and ducks

**Fig. 7:**
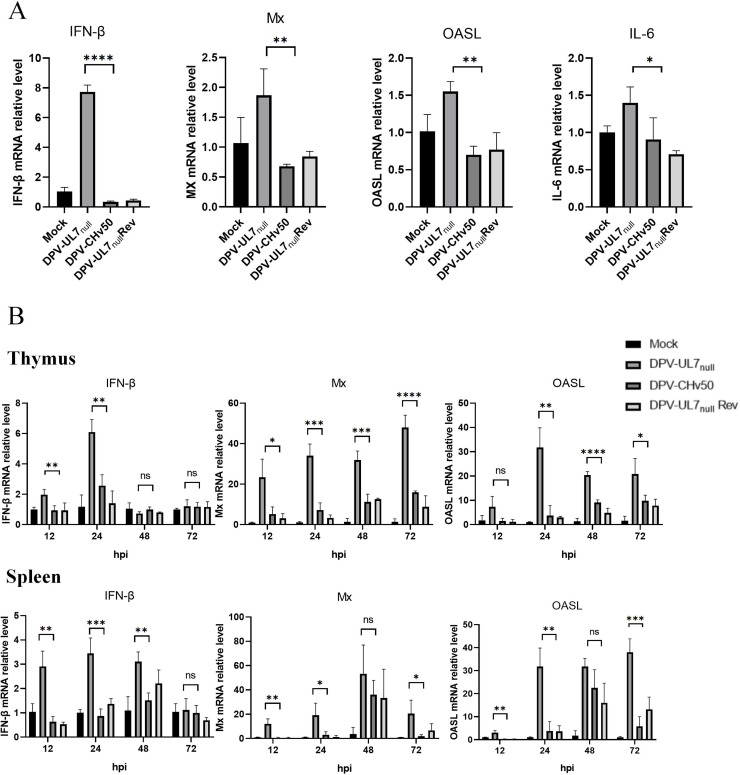
The DPV UL7 protein deficiency increases immune responses in DEFs and ducks. (A) DEFs were infected with DPV-UL7_null_, DPV-UL7_null_ Rev, or DPV-CHv50 at an MOI of 0.01. At 24 h postinfection (hpi), the cells were collected, and the mRNA expression levels of IFN-β, IL-6, Mx, and OASL were measured by RT‒qPCR. (B) Fourteen-day-old ducklings were infected with 100 TCID_50_ of DPV-UL7_null_, DPV-UL7_null_ Rev, or DPV-CHv50. Spleen and thymus tissues were collected at 12, 24, 36, and 48 hpi, and the mRNA levels of IFN-β, Mx, and OASL were analysed by RT‒qPCR. The data were analysed by one-way ANOVA (*P < 0.1, **P < 0.01, ***P < 0.001, ****P < 0.0001; ns indicates no significant difference).

Building on previous findings that the UL7 protein antagonizes host innate immunity, we designed *in vitro* and *in vivo* experiments to validate this mechanism. DEFs and 14-day-old ducklings were infected with DPV-UL7_null_, DPV-UL7_null_ Rev, or DPV-CHv50 at infection doses of an MOI of 0.01 (cells) and a TCID_50_ of 100 (ducklings). The qRT‒PCR results (Fig. 7A and B) revealed that at various time points postinfection, the transcription levels of key antiviral factors—IFN-β and its downstream effector molecules Mx and OASL—were significantly greater in the DPV-UL7_null_ group than in the other two groups. In contrast, the DPV-UL7_null_ Rev and DPV-CHv50 groups maintained relatively low expression levels of these immune factors. These results not only confirm previous findings but also clearly demonstrate that the UL7 protein effectively suppresses host IFN-β signalling pathway activation, thereby reducing the expression of ISGs, such as Mx and OASL. This immune evasion mechanism likely serves as a critical molecular basis by which UL7 promotes viral proliferation, explaining how *UL7* deficiency leads to diminished viral replication capacity.

### UL7 protein deficiency inhibits pathogenicity in ducklings

**Fig. 8:**
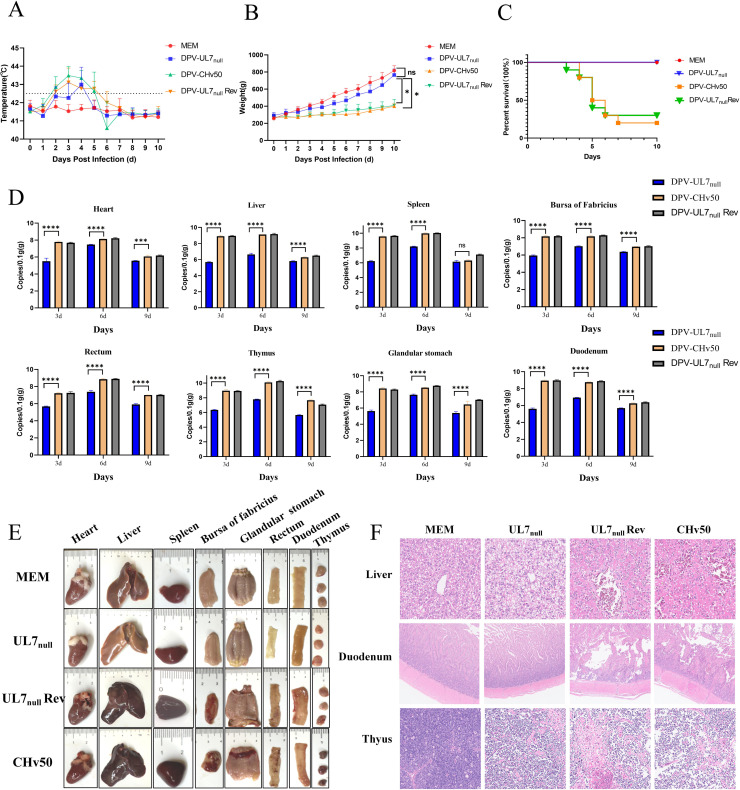
UL7 protein deficiency suppresses pathogenicity in ducklings. (A) Fourteen-day-old ducklings were inoculated with 10^6^ TCID_50_ of DPV-UL7_null_, DPV-UL7_null_ Rev, or DPV-CHv50 and monitored for 10 days to record changes in body temperature, (B) weight gain, and (C) survival rates. (D) Necropsy was performed on day 6 postinfection (dpi) to compare visceral lesions among the groups. (E) Viral DNA loads in selected tissues were quantified by RT‒qPCR using UL30 gene-specific primers to measure the number of viral genome copies. The data were analysed by one-way ANOVA (***P < 0.001, ****P < 0.0001; ns indicates no significant difference).

To evaluate the impact of the UL7 protein on DPV pathogenicity, we infected 14-day-old ducklings with 10^6^ TCID_50_ DPV-UL7_null_, DPV-UL7_null_ Rev, or DPV-CHv50 and then continuously observed the ducklings for 10 days. The DPV-UL7_null_ group showed no mortality within 10 days (100 % survival rate), exhibited only transient fever, and demonstrated body weight gain that was comparable to that of the MEM control group. In contrast, the DPV-UL7_null_ Rev and DPV-CHv50 groups presented prolonged fever (Fig. 8A), significantly slower weight gain (Fig. 8B), and varying degrees of mortality, with survival rates of only 30 % and 20 %, respectively (Fig. 8C). qPCR analysis of viral loads in various tissues revealed that the DPV-UL7_null_ strain presented significantly lower replication levels than both the parental and revertant strains at most time points and in all the examined organs (Fig. 8D). Overall, the viral load in DPV-UL7_null_-infected ducks was markedly lower than that in the DPV-UL7_null_ Rev and DPV-CHv50 groups, which contributed to the lower mortality and morbidity observed in this group.

To further investigate the pathogenic characteristics of DPV-UL7_null_, we performed necropsies on ducklings from all three groups at 6 days postinfection. Pathological examination revealed that the DPV-UL7_null_ group exhibited only mild thymic haemorrhage, with no significant lesions in other organs; these findings were similar to those of the MEM control group. In contrast, the DPV-UL7_null_ Rev and DPV-CHv50 groups presented severe multiorgan pathological damage, including hepatic congestion and necrosis, splenic congestion and swelling, thymic and bursal congestion and atrophy, thinning and haemorrhage in the duodenum and caecum, and notably, a characteristic haemorrhagic band at the proventriculus‒gizzard junction (Fig. 8E). Histopathological analysis further confirmed significant lesions in the liver, duodenum, and thymus of both the DPV-UL7_null_ Rev and DPV-CHv50 infection groups, including hapatocellular degeneration and necrosis, disordered hepatic cord structures; duodenal hemorrhage and necrosis, necrotic shedding of intestinal villi and hamorrhagic necrosis in thymic tissue. In contrast,in the DPV-UL7_null_ infection group, only mild pathological changes in thymic tissue were induced, with no observable histological damage to the liver or duodenum. The MEM group presented intact organ structures with no pathological alterations observed in any of the tissues (Fig. 8F).These findings demonstrate that the UL7 protein contributes to DPV pathogenicity and that its deletion attenuates the virulence of DPV.

## Discussion

The innate immune system is the first line of defence against pathogen invasion. The innate immune system recognizes PAMPs through PRRs, which then activate downstream signalling pathways and induce the production of broad-spectrum antiviral type I interferons (IFNs), which play a central role in antiviral immune responses (Kowalinski et al., 2011; Paludan et al., 2011). Herpesviruses are pathogens with sophisticated immune evasion strategies, and these viruses have evolved multiple mechanisms to counteract host innate immunity; these mechanisms are critical for establishing persistent infection and completing the replication cycle (Zheng, 2018). Studies have shown that DPV can modulate host innate immune responses via various viral proteins, including UL41 (98; He et al., 2022), UL24 (Ruan et al., 2024), UL47 (T. He et al., 2020), US3 (Liu et al., 2022), and VP16 (100), during the late stages of infection, and all of these proteins have been confirmed to participate in this process. Although significant progress has been made in elucidating the molecular mechanisms by which DPV antagonizes host innate immunity and revealing the key roles of multiple viral proteins in immune evasion, a systematic understanding of the activation mechanisms, signalling pathways, and regulatory networks of the host innate immune response to DPV infection is lacking.

This study focused on the molecular mechanism by which the DPV UL7 protein regulates host innate immune responses. Previous research has demonstrated that UL7 can influence IFN-β promoter activity (Gao et al., 2022), and its homologue UL103 interacts with various cellular antiviral proteins, including IFI16; these findings suggest that UL7 may perform similar immunomodulatory functions (Ortiz et al., 2016). However, the specific molecular mechanisms by which herpesvirus UL7 proteins regulate host immune responses remain unexplored. This study is the first to reveal that DPV UL7 significantly suppresses the poly(I:C)-induced expression of IFN-β and its downstream effector molecules Mx and OASL while also markedly inhibiting the IFN-β promoter activity that is induced by both the cGAS-STING and RIG-I/MDA5 signalling pathways. This finding expands the known family of viral proteins that are capable of simultaneously blocking DNA- and RNA-sensing pathways. Mechanistically, although the UL7 protein downregulates the mRNA levels of TBK1 and IRF7, it does not significantly affect their protein levels, suggesting the possible existence of posttranscriptional regulation or protein stability compensation mechanisms. Furthermore, UL7 can directly degrade the RIG-I protein, leading to reduced activity of the RIG-I–MAVS signalling pathway, which may be a key reason for its indirect suppression of downstream TBK1/IRF7 transcription. However, the precise molecular mechanisms by which UL7 regulates TBK1 and IRF7 expression still require further experimental validation.

Although the cGAS–STING DNA signalling pathway has been extensively studied, the mechanisms by which the RIG-I–MDA5 signalling pathway is regulated remain unclear. The novelty of the findings of this study lies in the demonstration, for the first time, that UL7 promotes RIG-I protein degradation via the proteasome or lysosome pathway, thereby suppressing RIG-I/MDA5 signalling pathway activation. These findings provide new theoretical insights into the molecular mechanisms by which herpesviruses evade host RNA recognition systems.

Deletion of the *UL7* gene significantly impaired DPV replication efficiency, indicating that this protein may play a crucial role in the viral life cycle. By analysing key stages of the herpesvirus life cycle—including viral attachment, invasion, and genome replication—this study demonstrated, for the first time, that while *UL7* deletion does not affect early infection processes, it markedly suppresses viral particle release and cell-to-cell spread. This finding suggest that UL7 primarily promotes viral proliferation by facilitating late-stage virion release and dissemination. The successful rescue of the UL7-deficient virus confirmed that UL7 is not essential for DPV replication, but its deletion significantly reduces viral proliferation in cells. This finding is consistent with the characteristics of UL7 homologues in other herpesviruses, such as HSV-1 and PRV, where deletion of *UL7* (or its orthologues ORF42 and UL103) similarly reduces viral titres to varying degrees (Ahlqvist & Mocarski, 2011; Butnaru & Gaglia, 2019; Das et al., 2014; Fuchs et al., 2005; Xu et al., 2016). Additionally, multiple studies have indicated that herpesvirus UL7 family proteins participate in secondary envelopment and play a critical role in viral egress (Ahlqvist & Mocarski, 2011; Albecka et al., 2017; Burridge et al., 1988; Butt et al., 2020; Fuchs et al., 2005; H. P. He et al., 2020; Schmitt & Keil, 1996; Zaidel-Bar & Geiger, 2010). Our results are largely consistent with the functional features of α-herpesvirus UL7 proteins, further suggesting that DPV UL7 plays a conserved role within the Herpesviridae family.

Research has shown that in α-herpesviruses, including HSV-1, HSV-2, and PRV, UL7 proteins are closely associated with viral virulence, and the deletion of these proteins significantly attenuates pathogenicity (Fuchs et al., 2005; Shibazaki et al., 2020; Xu et al., 2017), highlighting the critical role of these proteins in α-herpesvirus pathogenesis. In this study, the *UL7*-deficient strain DPV-UL7_null_ caused reduced morbidity and mortality, exhibited weakened replication in multiple organs, and induced only localized thymic lesions without causing significant damage to other tissues. These findings collectively demonstrate that *UL7* deletion markedly diminishes DPV pathogenicity in ducks.

In summary, this study revealed for the first time that UL7 suppresses IFN-β production and antagonizes host innate immunity by targeting RIG-I for degradation while also promoting viral release and cell-to-cell spread to increase viral proliferation. UL7 is a conserved herpesvirus virulence factor, and its deletion significantly attenuates viral pathogenicity, suggesting its potential for use as a target for the development of live attenuated vaccines. These findings not only elucidate the molecular mechanisms by which DPV evades host innate immunity but also provide new insights into the pathogenesis of efficient viral replication and persistent infection in ducks.

## CRediT authorship contribution statement

**Xing Lan:** Data curation, Formal analysis, Methodology, Writing – original draft, Writing – review & editing. **Yuan yuan Hao:** Data curation, Methodology, Software, Writing – review & editing. **Mingshu Wang:** Funding acquisition, Investigation, Methodology, Project administration, Supervision, Validation, Visualization. **Linjiang Yang:** Resources, Software, Supervision. **Liping Wu:** Investigation, Methodology, Resources. **Anchun Cheng:** Data curation, Funding acquisition, Project administration, Validation.

## Disclosures

The authors declare that the research was conducted in the absence of any commerciaor financial relationships that could be construed as a potential conflict of interest
